# Infectious disease consultations in a Japanese tertiary care teaching hospital: a retrospective review of 508 cases

**DOI:** 10.1186/s12913-018-3802-6

**Published:** 2018-12-19

**Authors:** Yoshiro Hadano, Takanori Matsumoto

**Affiliations:** 1 Department of Infectious Diseases, St. Mary’s Hospital, 422 Tsubukuhonmachi, Kurume, Fukuoka, 830-8543 Japan; 2 Biostatistics Center, Kurume University School of Medicine, Kurume, Japan; 3 Department of Pharmacy, St. Mary’s Hospital, Kurume, Japan

**Keywords:** Infectious diseases, Consultation services, Japan, Surgery

## Abstract

**Objective:**

Limited epidemiological data are available at tertiary care teaching hospitals in Japan. We reviewed infectious disease (ID) consultations in a tertiary acute care teaching hospital in Japan.

**Methods:**

This is a retrospective review of the ID consultations from October 2016 to December 2017. The demographic data, such as requesting department, consultation wards, and final diagnosis, were analyzed.

**Results:**

There were 508 ID consultations during the 15-month study period. Among the 508 consultations, 201 cases (39.6%) were requested from the internal medicine department and 307 cases (60.4%) were requested from departments other than internal medicine. The most frequent requesting departments were Surgery (*n* = 102, 20.1%), Pulmonary Medicine (*n* = 41, 8.1%), and Plastic Surgery (*n* = 35, 6.7%). The most common diagnoses were intra-abdominal (*n* = 81, 16.0%), respiratory (*n* = 62, 12.2%), and skin and soft tissue infections (*n* = 59, 11.6%). ID consultations for disease diagnosis and management were more frequent in the internal medicine group than in the non-internal medicine group (37 cases, 20.8% vs. 40 cases, 13.7%, *p* = 0.046), and the number of requests for consultations for noninfectious diseases at the time of final diagnosis was higher in the internal medicine group than in the non-internal medicine group (21 cases, 11.8% vs. 16 cases, 5.5%, *p* = 0.0153).

**Conclusion:**

Some physicians prefer ID specialists to identify and solve various medical problems. Internists had a greater tendency to request consultations for diagnostic problems, and noninfectious disease specialists have more requests for consultation at the point of final diagnosis. The role of ID specialists is expanding, from individual patient management to antibiotic stewardship, antibiotic prophylaxis, and development of and adherence to antibiotic protocol implementation based on the hospital’s microbial susceptibility and infection control. Although the number of specialists is limited in Japan, ID services now play an important role for achieving a good outcome in patient management.

## Introduction

Infectious disease (ID) specialists are important in medical care but are a rare subspecialty in Japan. There are only 0.9 ID specialists per 100, 000 population in Japan compared to 2.4 ID specialists per 100, 000 population in the United States [1].

According to a previous report, there were only 1140 board-certified ID specialists in Japan [2]. The Japanese Association for Infectious Diseases (JAID) has imposed 3 years of postgraduate training at accredited programs or collaborative programs to qualify for the examination, and 57 physicians passed the ID board examination in 2012 [1]. As a result, most hospitals have no clinical ID specialists, including large tertiary care hospitals, especially in a regional or rural area in Japan. Therefore, some ID fellows have moved to other hospitals after their ID fellowship and launched a new ID division there. For most of these cases, there is initially only one clinical ID specialist in a new hospital. Approximately 800 institutions have ID coverage in Japan (JAID data). Although ID consultation data are published at a comprehensive cancer center in Japan [2], there are limited epidemiological data at tertiary care teaching hospitals in Japan. In October 2016, one board-certified clinical ID specialist who had finished a clinical ID fellowship in Japan came to St. Mary’s Hospital and launched a northwestern-style ID consultation service. The objective of this study is to report our experience in establishing an ID consultation service and to describe the value of an ID specialist in providing consultations. We further determined the skills and knowledge required for Japanese ID specialists at a tertiary acute care teaching hospital in Japan.

## Methods

St. Mary’s Hospital (Kurume, Japan) is a nonuniversity, 1097-bed tertiary acute care teaching hospital. This hospital is located in a regional hub city in southwestern Japan. The hospital does not have a Department of General Internal Medicine, and the Department of Emergency Medicine is sometimes in charge of patients who either need critical care or have multisystem problems. In this single-center retrospective study, we analyzed the ID consultation database from October 2016 to December 2017. All formal ID consultations, including pediatrics, were eligible for analysis, and informal (so-called “curbside”) consultations were excluded. The following demographic data of the study participants were collected: age, sex, requesting department, the reasons for referral at the time of the first ID consultation, where the consultation took place (intensive care unit, general ward, outpatient/emergency room), presence or absence of bacteremia, final diagnosis, and 30-day mortality. The initial reasons for the ID consultation were categorized as diagnosis and management (fever or elevated inflammatory markers, such as white blood cell count and C-reactive protein, of unknown etiology, suspicion of infections, positive blood culture), treatment of established infections (management of infections already diagnosed such as intra-abdominal infections, respiratory infections, urinary tract infection, and so on), infection control (isolation of opportunistic or multidrug-resistant pathogens, suspicion of tuberculosis, others), and surgical antimicrobial prophylaxis.

This study was approved by the St. Mary’s Hospital Research Ethics Committee (No. 17–0201). ID consultations are a part of the standard patient care. Since this was an observational study, requirement for informed consent was waived.

### Statistical analysis

Categorical data were analyzed using either the chi-squared test or Fisher’s exact test, and non-categorical data were analyzed using either the Student’s T-test or Mann-Whitney U test. *P* values greater than 0.05 were considered statistically significant. All statistical analyses were performed using JMP Pro software program (version 13.0, SAS Institute, Cary, USA).

## Results

There were 508 ID consultations during the 15-month study period. Baseline characteristics of the patients are described in Table 1. The median age was 68 years (interquartile range, 55–79) (range 0–101 years). Twelve patients were less than 18 years old. Among the 508 consultations, 201 cases (39.6%) were requested from the internal medicine department and 307 cases (60.4%) were requested from staff not associated with internal medicine (non-internal medicine) department. After the cases of infection control and surgical antimicrobial prophylaxis were excluded, 470 cases were evaluated for the incidence of bacteremia and 30-day mortality. The incidence rates of bacteremia and 30-day mortality were 33.0% (155/470 cases) and 3.8% (19/470 cases), respectively. The departments that most frequently requested for consultations were the following: Surgery (*n* = 102, 20.1%), Pulmonary Medicine (*n* = 41, 8.1%), and Plastic Surgery (*n* = 35, 6.7%) (Table 2). In the final diagnosis, 471 (92.5%) cases were considered infectious diseases, and 37 cases (7.5%) were due to noninfectious causes. The most common diagnoses were intra-abdominal (*n* = 81, 16.0%), respiratory (*n* = 62, 12.2%), and skin and soft tissue infections (*n* = 59, 11.6%) (Table 3). Twelve cases involved isolation of opportunistic or multidrug-resistant pathogens, eight cases had a suspicion of tuberculosis, and eight cases had other causes. We examined the pattern of requests for ID consultations between internal medicine and non-internal medicine staff among 470 cases (Table 4). The ID setting and the incidence of bacteremia were similar between the two groups, but ID consultations for diagnosis and management were more frequent in the internal medicine group (37 cases, 20.8% vs. 40 cases, 13.7%, *p* = 0.046), and the incidence of requests for consultation by the non-internal medicine staff was higher at the final diagnosis of noninfectious diseases (21 cases, 11.8% vs. 16 cases, 5.5%, *p* = 0.015).

**Table 1:** Characteristics of the infectious disease consultations (*n* = 508)

Variables	No. of patients
Age (years)	68 (55–79)
Gender	
Male	282 (55.5%)
Female	226 (44.5%)
Consultation places	
General wards (1069 beds)	368 (72.4%)
Intensive care unit (28 beds)	101 (19.9%)
Outpatients/Emergency rooms	39 (7.7%)
Consult reasons	
Diagnosis	77 (15.2%)
Treatment	393 (77.4%)
Infection control	28 (5.5%)
Surgical antimicrobial prophylaxis	10 (1.9%)
Specialty	
Internal medicine	201 (39.6%)
(Cardiology, Diabetes and endocrinology, Gastroenterology, Hematology, Nephrology, Neurology, Respiratory, and Rheumatology)	
Non- Internal medicine	307 (60.4%)
(Cardiovascular surgery, Dermatology, Emergency Medicine, ENT, Gynecology, Neurosurgery, Orthopedic surgery, Psychiatry, Pediatrics, Plastic surgery, Surgery, Thoracic surgery, and Urology)	
Bacteremia	155/470 (33.0%)
30-day mortality	19/470 (3.8%)

**Table 2:** Characteristics of the infectious diseases consultations (*n* = 508)

Hospital departments	No. of patients
Surgery	102 (20.1%)
Respiratory	41 (8.1%)
Plastic surgery	35 (6.7%)
Orthopedic surgery	34 (6.7%)
Emergency Medicine	32 (6.3%)
Cardiovascular surgery	32 (6.3%)
Neurology	29 (5.7%)
Gastroenterology	28 (5.5%)
Hematology	26 (5.1%)
Nephrology	24 (4.7%)
Dermatology	22 (4.3%)
Gynecology	21 (4.1%)
Neurosurgery	20 (3.9%)
Cardiology	13 (2.6%)
Diabetes and endocrinology	13 (2.6%)
Rheumatology	11 (2.2%)
Psychiatry	11 (2.2%)
ENT	7 (1.4%)
Others	7 (1.4%)
Total	508 (100%)

Others: Pediatrics, Thoracic surgery, Urology

**Table 3:** Final diagnosis of infectious diseases (ID) by ID specialists (*n* = 508)

Variables	No. of patients
Intra-abdominal infections	81 (16.0%)
Respiratory infections	62 (12.2%)
Skin and soft tissue infections	59 (11.6%)
Genitourinary infections	48 (9.4%)
Bone/Joint infections	46 (9.1%)
Non-infectious diseases	37 (7.5%)
Bacteremia	31 (6.1%)
Infection control	28 (5.5%)
Cardiovascular infections	20 (3.9%)
CNS infections	18 (3.5%)
CRBSI	15 (3.0%)
Hepatobiliary tract infections	15 (3.0%)
Surgical antimicrobial prophylaxis	9 (1.7%)
Febrile neutropenia	8 (1.6%)
HEENT infections	7 (1.3%)
Unknown	5 (0.9%)
Others	19 (3.7%)
Total	508 (100%)

**Table 4:** Pattern of infectious disease consultations between internal medicine and non-internal medicine departments (*n* = 470)

Variables	Internal medicine (*n* = 178) (%)	Non-internal medicine (*n* = 292) (%)	*P*-value
Setting			
General wards	135 (75.8)	208 (71.2)	
Intensive care unit	30 (16.9)	64 (21.9)	0.412
Outpatients/Emergency rooms	13 (7.3)	20 (6.9)	
Bacteremia cases	67 (37.6)	88 (30.4)	0.0945
Consultation request			
Diagnosis and management	37 (20.8)	40 (13.7)	0.0463
Treatment of established infections	141 (79.2)	252 (86.3)	
Final diagnosis			
Infectious diseases	157 (88.2)	276 (94.5)	0.0153
Non-infectious diseases	21 (11.8)	16 (5.5)	

## Discussion

We evaluated 508 ID consultations in a newly established ID consultation department at a Japanese tertiary care hospital. Our study demonstrates that most of our departments referred to ID specialists for consultations during this study period. The number of ID consultations was higher in the non-internal medicine group compared to that in the internal medicine group. The departments most commonly requesting this service were Surgery, Plastic Surgery, Orthopedic Surgery, and Cardiovascular Surgery. According to a previous study at a comprehensive cancer center in Japan, it is suggested that many surgeons would prefer consultations from ID specialists [2]. Furthermore, the ID specialists had a tendency to assume a more direct role especially in the care of difficult cases [2]. In another report on the ideal relationship between physicians and consultants, the surgeons when compared to non-surgeons clearly wanted the consultant to be more involved in patient care and preferred a formal relationship rather than informal advice [3]. With increases in medical knowledge and training on infectious diseases, such as multidrug-resistant pathogens, new antibiotics, new diagnostic methods, new concepts for antimicrobial stewardship, and antimicrobial resistance, there have been changes in clinical practices for the treatment of infection. Thus, it may be difficult for physicians to continually update their knowledge for patient management of IDs. As a result, there might be increasing demand for an ID service similar to that in other countries. In this study, internists had a tendency to consult for more diagnostic problems, and both internists and non-internists had a similar need for consultation at the point of final diagnosis. The incidence rate of diagnostic consultation was 16%, which is lower than that in the previous studies [4–6], where the diagnostic consultations in difficult diagnostic cases were sometimes a challenge for the ID consultants. One of the roles of ID specialists is being a diagnostician. Some Japanese ID specialists learned clinical reasoning during ID fellowship and often have good diagnostic knowledge and skills. In Japan, diagnostic division, such as general internal medicine, is rare. Therefore, Japanese ID specialists are also required to diagnose nondiagnostic patients or patients who are difficult to diagnose, such as those with fever of unknown origin. Because our hospital has no diagnostic department, all of the physicians have a challenge to do the diagnostic workup in cases of unexplained fever or symptoms. ID specialists may help to care for patients who are difficult to diagnose especially in Japan [7, 8].

Similar to the situation in Japan, a report from Germany found that the ID services improved the quality of care and treatment outcome for IDs [4, 9, 10]. For example, ID consultation played a positive role in patient management resulting in a good outcome for patients with *Staphylococcus aureus* bacteremia. A previous meta-analysis demonstrated that bedside ID consultations are associated with better outcomes, including all-cause 30-day mortality, which declined from 26 to 12% in favor of the ID consultation group [relative risk, 0.53 (95% CI, 0.43–0.65)] [11]. Most studies reported a survival benefit in the adjusted analysis [12]. However, there are not enough ID specialists in Japan. As of 2013, there were only 1140 board-certified ID specialists in Japan [1]. In Japan, early diagnosis and treatment by an ID specialist is important because of the unmet need and limited number of these specialists compared to that in other developed countries where an adequate number of ID specialists are able to contribute to improving the medical outcome.

Japanese hospital deans usually require ID specialists to work as infection control practitioners [13]. In our study, most of the consultations are case-based, but a consultation for the suspicion of tuberculosis is more common. Although this is a unique skill, Japanese ID specialists should be adaptable and learn as much as possible during their ID fellowship training.

Our study had several limitations related to its retrospective and uncontrolled nature. We cannot exclude classification biases or diagnostic errors associated with the evaluation performed by the ID specialists. Secondly, this retrospective study was conducted at a single center, and the results may not apply to other settings with different consultation styles. Thirdly, we could not show the proportion of ID consultations for all infectious diseases in each department. Finally, our result did not include informal consultations, which have a role in our workload. In our opinion, curbside consultations are an important issue, especially in situations where the number of ID specialists is small [14]. In cases of curbside consultation, overlooking of important information sometimes happens, leading to false diagnoses; thus, we often keep it in general answer. In individual cases, which are difficult to diagnose, we recommend formal ID consultation. Nevertheless, our study is valuable as it describes the pattern of ID consultations in a tertiary care hospital in Japan. Further research is needed to determine the value of ID specialists in Japan, which is a relatively new consultation service.

## Conclusion

In conclusion, we described the roles of ID specialists at an acute tertiary care teaching hospital. The surgical department was the most dependent on ID specialist consultations. Internists had a tendency to consult more for diagnostic problems, while non-internists sought consultations more often at the point of final diagnosis. The role of ID specialists is expanding, from individual patient management to antibiotic stewardship, antibiotic prophylaxis, and development of and adherence to antibiotic protocol implementation based on the hospital’s microbial susceptibility and infection control. ID specialists also educate their hospital’s physicians. Although the number of ID specialists is limited, ID services play an important role in patient management to achieve a good outcome.

## Abbreviations

AMR: Antimicrobial resistance
CNS: Central nervous system
CRBSI: Catheter-related bloodstream infection
ENT: Ear, Nose, and Throat
HEENT: Head, Eyes, Ears, Nose, and Throat
ICU: Intensive care unit
ID: Infectious disease
IQR: Interquartile range
